# 5-Iodo-2,7-dimethyl-3-(4-methyl­phenyl­sulfon­yl)-1-benzofuran

**DOI:** 10.1107/S1600536812037932

**Published:** 2012-09-08

**Authors:** Hong Dae Choi, Pil Ja Seo, Uk Lee

**Affiliations:** a Department of Chemistry, Dongeui University, San 24 Kaya-dong, Busanjin-gu, Busan 614-714, Republic of Korea; b Department of Chemistry, Pukyong National University, 599-1 Daeyeon 3-dong, Nam-gu, Busan 608-737, Republic of Korea

## Abstract

In the title compound, C_17_H_15_IO_3_S, the 4-methyl­phenyl ring makes a dihedral angle of 76.95 (5)° with the mean plane [r.m.s. deviation = 0.019 (2) Å] of the benzofuran fragment. In the crystal, mol­ecules are linked *via* pairs of C—H⋯O hydrogen bonds, forming inversion dimers. These dimers are connected by slipped π–π inter­actions between the benzene rings of neighbouring mol­ecules [centroid–centroid distance = 3.671 (3) Å and slippage = 1.049 (3) Å].

## Related literature

For background information and the crystal structures of related compounds, see: Choi *et al.* (2008 ▶); Seo *et al.* (2012 ▶).
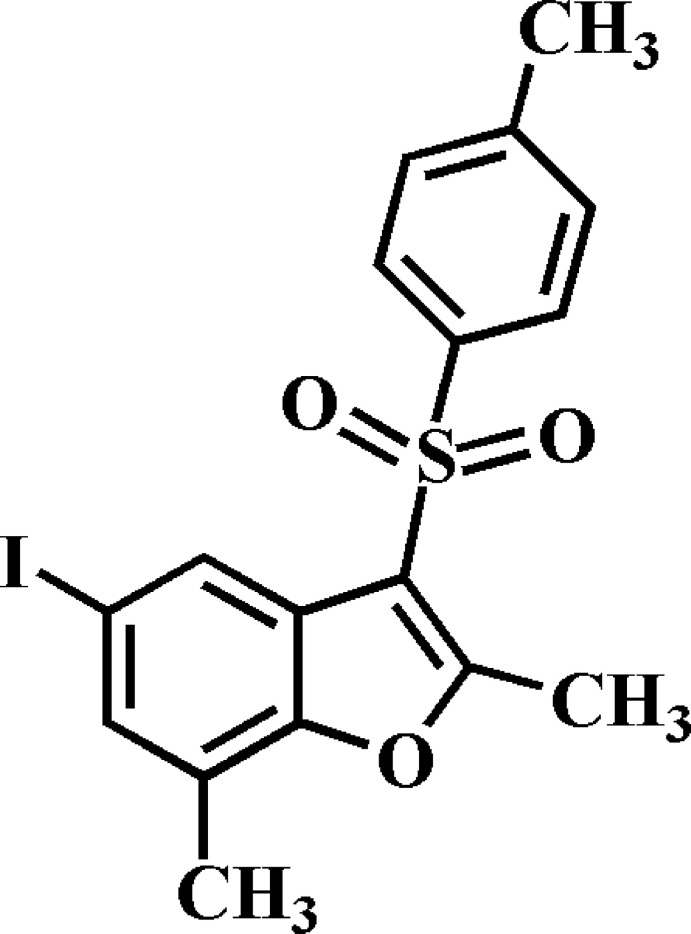


## Experimental

### 

#### Crystal data

C_17_H_15_IO_3_S
*M* *_r_* = 426.25
Monoclinic, 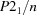

*a* = 11.5480 (5) Å
*b* = 9.9394 (4) Å
*c* = 14.8911 (6) Å
β = 107.611 (1)°
*V* = 1629.10 (12) Å^3^
*Z* = 4
Mo *K*α radiation
μ = 2.10 mm^−1^
*T* = 173 K
0.33 × 0.27 × 0.22 mm

#### Data collection

Bruker SMART APEXII CCD diffractometer
Absorption correction: multi-scan (*SADABS*; Bruker, 2009 ▶) *T*_min_ = 0.634, *T*_max_ = 0.746
16012 measured reflections
4075 independent reflections
3619 reflections with *I* > 2σ(*I*)
*R*_int_ = 0.028

#### Refinement

*R*[*F* ^2^ > 2σ(*F* ^2^)] = 0.027
*wR*(*F* ^2^) = 0.070
*S* = 1.05
4075 reflections
202 parameters
H-atom parameters constrained
Δρ_max_ = 0.49 e Å^−3^
Δρ_min_ = −0.91 e Å^−3^

### 

Data collection: *APEX2* (Bruker, 2009 ▶); cell refinement: *SAINT* (Bruker, 2009 ▶); data reduction: *SAINT*; program(s) used to solve structure: *SHELXS97* (Sheldrick, 2008 ▶); program(s) used to refine structure: *SHELXL97* (Sheldrick, 2008 ▶); molecular graphics: *ORTEP-3* (Farrugia, 1997 ▶) and *DIAMOND* (Brandenburg, 1998 ▶); software used to prepare material for publication: *SHELXL97*.

## Supplementary Material

Crystal structure: contains datablock(s) global, I. DOI: 10.1107/S1600536812037932/im2395sup1.cif

Structure factors: contains datablock(s) I. DOI: 10.1107/S1600536812037932/im2395Isup2.hkl

Supplementary material file. DOI: 10.1107/S1600536812037932/im2395Isup3.cml

Additional supplementary materials:  crystallographic information; 3D view; checkCIF report

## Figures and Tables

**Table 1:** Hydrogen-bond geometry (Å, °)

*D*—H⋯*A*	*D*—H	H⋯*A*	*D*⋯*A*	*D*—H⋯*A*
C16—H16⋯O3^i^	0.95	2.58	3.246 (2)	127

Symmetry code: (i) 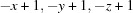.

**Fig. 1.**
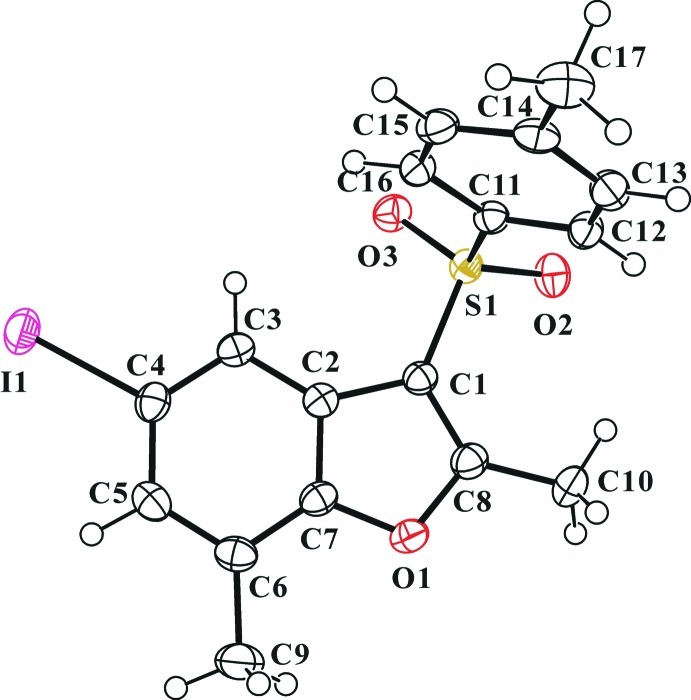
Molecular structure of the title compound with displacement ellipsoids drawn at the 50% probability level. H atoms are presented as small spheres of arbitrary radius.

**Fig. 2.**
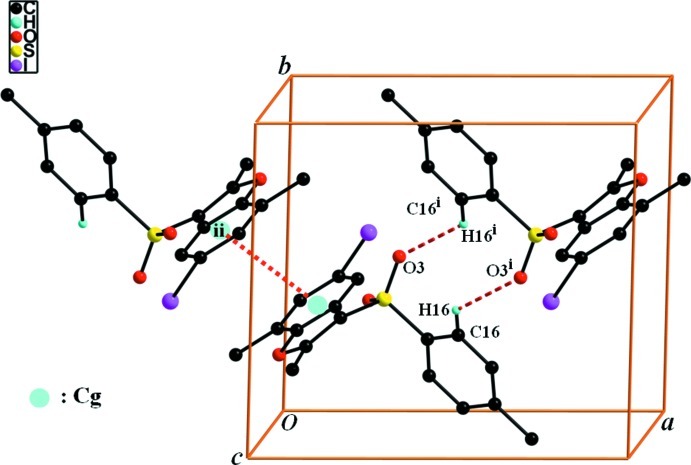
A view of the C—H···O and π–π interactions (dotted lines) in the crystal structure of the title compound. H atoms not participating in hydrogen-bonding were omitted for clarity. [Symmetry codes: (i) - x + 1, - y + 1, - z + 1; (ii) - x, - y + 1, - z + 1.]

## supplementary crystallographic information

### Comment

As a part of our ongoing study of 5-iodo-2,7-dimethyl-benzofuran derivatives containing either phenyl-sulfonyl (Choi *et al.*, 2008) or 4-fluorophenyl-sulfonyl (Seo *et al.*, 2012) substituents in 3-position, we report herein the crystal structure of the title compound.

In the title molecule (Fig. 1), the benzofuran unit is essentially planar, with a mean deviation of 0.019 (2) Å from the least-squares plane defined by the nine constituent atoms. The dihedral angle between the 4-methylphenyl ring and the mean plane of the benzofuran fragment is 76.95 (5)°. In the crystal structure, molecules are linked via pairs of C—H···O hydrogen bonds (Fig. 2 & Table 1), forming inversion dimers. These dimers are connected by slipped π–π interactions between the benzene rings of neighbouring molecules, with a Cg···Cg^ii^ distance of 3.671 (3) Å and an interplanar distance of 3.518 (2) Å resulting in a slippage of 1.049 (3) Å (Fig. 2, Cg is the centroid of the C2–C7 benzene ring).

### Experimental

3-Chloroperoxybenzoic acid (77%, 381 mg, 1.7 mmol) was added in small portions to a stirred solution of 5-iodo-2,7-dimethyl-3-(4-methylphenylsulfanyl)-benzofuran (315 mg, 0.8 mmol) in dichloromethane (50 mL) at 273 K. After being stirred at room temperature for 10h, the mixture was washed with saturated sodium bicarbonate solution, the organic layer was separated and dried over magnesium sulfate. After filtration the solution was concentrated at reduced pressure. The residue was purified by column chromatography (benzene ) to afford the title compound as a colorless solid [yield 68%, m.p. 483–484 K; *R*_f_ = 0.56 (benzene)]. Single crystals suitable for X-ray diffraction were prepared by slow evaporation of a solution of the title compound in acetone at room temperature.

### Refinement

All H atoms were positioned geometrically and refined using a riding model, with C—H = 0.95 Å for aryl and 0.98 Å for methyl H atoms. *U*_iso_(H) = 1.2*U*_eq_(C) for aryl and 1.5*U*_eq_(C) for methyl H atoms. The positions of methyl hydrogens were optimized rotationally.

### Crystal data

**Table:**

C_17_H_15_IO_3_S	*F*(000) = 840
*M**_r_* = 426.25	*D*_x_ = 1.738 Mg m^−^^3^
Monoclinic, *P*2_1_/*n*	Mo *K*α radiation, λ = 0.71073 Å
Hall symbol: -P 2yn	Cell parameters from 7311 reflections
*a* = 11.5480 (5) Å	θ = 2.5–28.3°
*b* = 9.9394 (4) Å	µ = 2.10 mm^−^^1^
*c* = 14.8911 (6) Å	*T* = 173 K
β = 107.611 (1)°	Block, colourless
*V* = 1629.10 (12) Å^3^	0.33 × 0.27 × 0.22 mm
*Z* = 4	

### Data collection

**Table:**

Bruker SMART APEXII CCD diffractometer	4075 independent reflections
Radiation source: rotating anode	3619 reflections with *I* > 2σ(*I*)
Graphite multilayer monochromator	*R*_int_ = 0.028
Detector resolution: 10.0 pixels mm^-1^	θ_max_ = 28.4°, θ_min_ = 2.0°
φ and ω scans	*h* = −15→15
Absorption correction: multi-scan (*SADABS*; Bruker, 2009)	*k* = −11→13
*T*_min_ = 0.634, *T*_max_ = 0.746	*l* = −19→19
16012 measured reflections	

### Refinement

**Table:**

Refinement on *F*^2^	Primary atom site location: structure-invariant direct methods
Least-squares matrix: full	Secondary atom site location: difference Fourier map
*R*[*F*^2^ > 2σ(*F*^2^)] = 0.027	Hydrogen site location: difference Fourier map
*wR*(*F*^2^) = 0.070	H-atom parameters constrained
*S* = 1.05	*w* = 1/[σ^2^(*F*_o_^2^) + (0.0382*P*)^2^ + 0.5412*P*] where *P* = (*F*_o_^2^ + 2*F*_c_^2^)/3
4075 reflections	(Δ/σ)_max_ = 0.001
202 parameters	Δρ_max_ = 0.49 e Å^−^^3^
0 restraints	Δρ_min_ = −0.91 e Å^−^^3^

### Special details

**Table:**

Geometry. All esds (except the esd in the dihedral angle between two l.s. planes) are estimated using the full covariance matrix. The cell esds are taken into account individually in the estimation of esds in distances, angles and torsion angles; correlations between esds in cell parameters are only used when they are defined by crystal symmetry. An approximate (isotropic) treatment of cell esds is used for estimating esds involving l.s. planes.
Refinement. Refinement of F^2^ against ALL reflections. The weighted R-factor wR and goodness of fit S are based on F^2^, conventional R-factors R are based on F, with F set to zero for negative F^2^. The threshold expression of F^2^ > 2sigma(F^2^) is used only for calculating R-factors(gt) etc. and is not relevant to the choice of reflections for refinement. R-factors based on F^2^ are statistically about twice as large as those based on F, and R- factors based on ALL data will be even larger.

### Fractional atomic coordinates and isotropic or equivalent isotropic displacement parameters (Å^2^)

**Table:**

	*x*	*y*	*z*	*U*_iso_*/*U*_eq_	
I1	0.279119 (14)	0.635214 (15)	0.727688 (10)	0.03911 (7)	
S1	0.28800 (5)	0.38147 (4)	0.33837 (3)	0.02292 (10)	
O1	0.02336 (13)	0.22668 (14)	0.42013 (11)	0.0294 (3)	
O2	0.23919 (16)	0.36296 (14)	0.23798 (11)	0.0318 (3)	
O3	0.32866 (14)	0.51329 (13)	0.37468 (10)	0.0294 (3)	
C1	0.17945 (18)	0.33310 (19)	0.39126 (14)	0.0235 (4)	
C2	0.17346 (18)	0.37898 (17)	0.48211 (14)	0.0230 (4)	
C3	0.23659 (18)	0.47147 (19)	0.54969 (14)	0.0258 (4)	
H3	0.3051	0.5191	0.5435	0.031*	
C4	0.1941 (2)	0.4900 (2)	0.62619 (14)	0.0278 (4)	
C5	0.0950 (2)	0.4200 (2)	0.63774 (15)	0.0303 (4)	
H5	0.0701	0.4369	0.6920	0.036*	
C6	0.03155 (19)	0.3262 (2)	0.57189 (15)	0.0290 (4)	
C7	0.07526 (18)	0.31046 (19)	0.49528 (14)	0.0253 (4)	
C8	0.08764 (19)	0.2427 (2)	0.35754 (15)	0.0277 (4)	
C9	−0.0757 (2)	0.2491 (3)	0.58031 (19)	0.0416 (5)	
H9A	−0.1400	0.2503	0.5197	0.062*	
H9B	−0.1058	0.2906	0.6286	0.062*	
H9C	−0.0518	0.1560	0.5981	0.062*	
C10	0.0456 (2)	0.1610 (2)	0.27101 (18)	0.0397 (5)	
H10A	0.0914	0.1860	0.2278	0.059*	
H10B	−0.0412	0.1772	0.2406	0.059*	
H10C	0.0587	0.0654	0.2872	0.059*	
C11	0.40852 (17)	0.26776 (18)	0.38089 (13)	0.0222 (4)	
C12	0.4107 (2)	0.15132 (19)	0.32984 (14)	0.0260 (4)	
H12	0.3491	0.1343	0.2722	0.031*	
C13	0.5049 (2)	0.0601 (2)	0.36479 (15)	0.0288 (4)	
H13	0.5070	−0.0200	0.3305	0.035*	
C14	0.59577 (19)	0.0837 (2)	0.44849 (15)	0.0277 (4)	
C15	0.59026 (19)	0.2003 (2)	0.49941 (15)	0.0279 (4)	
H15	0.6511	0.2167	0.5576	0.033*	
C16	0.49679 (19)	0.2923 (2)	0.46593 (14)	0.0257 (4)	
H16	0.4933	0.3712	0.5009	0.031*	
C17	0.6981 (2)	−0.0151 (2)	0.48381 (19)	0.0383 (5)	
H17A	0.6676	−0.1066	0.4671	0.057*	
H17B	0.7315	−0.0075	0.5524	0.057*	
H17C	0.7620	0.0044	0.4549	0.057*	

### Atomic displacement parameters (Å^2^)

**Table:**

	*U*^11^	*U*^22^	*U*^33^	*U*^12^	*U*^13^	*U*^23^
I1	0.03727 (11)	0.04423 (10)	0.03268 (10)	−0.00198 (6)	0.00588 (7)	−0.01371 (6)
S1	0.0247 (2)	0.0226 (2)	0.0205 (2)	−0.00060 (17)	0.00545 (19)	0.00125 (16)
O1	0.0244 (7)	0.0299 (7)	0.0336 (8)	−0.0057 (6)	0.0082 (6)	−0.0044 (6)
O2	0.0369 (9)	0.0364 (8)	0.0192 (7)	0.0038 (6)	0.0040 (7)	0.0024 (5)
O3	0.0339 (8)	0.0217 (6)	0.0328 (8)	−0.0032 (6)	0.0106 (7)	0.0010 (5)
C1	0.0213 (9)	0.0236 (8)	0.0248 (9)	0.0005 (7)	0.0058 (8)	0.0001 (7)
C2	0.0209 (9)	0.0219 (8)	0.0249 (9)	0.0031 (7)	0.0052 (8)	0.0016 (7)
C3	0.0222 (10)	0.0263 (9)	0.0278 (10)	−0.0010 (7)	0.0058 (8)	0.0002 (7)
C4	0.0268 (10)	0.0294 (9)	0.0238 (10)	0.0019 (8)	0.0027 (8)	−0.0011 (7)
C5	0.0296 (11)	0.0363 (10)	0.0268 (10)	0.0041 (9)	0.0113 (9)	0.0037 (8)
C6	0.0239 (10)	0.0313 (10)	0.0319 (11)	0.0017 (8)	0.0087 (9)	0.0058 (8)
C7	0.0220 (10)	0.0230 (9)	0.0290 (10)	0.0001 (7)	0.0047 (8)	0.0004 (7)
C8	0.0242 (10)	0.0264 (9)	0.0315 (11)	0.0000 (8)	0.0068 (8)	−0.0023 (8)
C9	0.0330 (13)	0.0508 (14)	0.0434 (13)	−0.0100 (10)	0.0153 (11)	0.0022 (11)
C10	0.0372 (13)	0.0400 (12)	0.0421 (13)	−0.0113 (10)	0.0124 (11)	−0.0185 (10)
C11	0.0215 (9)	0.0230 (8)	0.0221 (9)	−0.0015 (7)	0.0063 (7)	0.0015 (7)
C12	0.0273 (10)	0.0271 (9)	0.0226 (9)	−0.0037 (8)	0.0062 (8)	−0.0027 (7)
C13	0.0328 (11)	0.0248 (9)	0.0314 (10)	−0.0008 (8)	0.0137 (9)	−0.0004 (7)
C14	0.0249 (10)	0.0277 (9)	0.0332 (11)	−0.0017 (8)	0.0127 (9)	0.0070 (8)
C15	0.0222 (10)	0.0345 (10)	0.0250 (10)	−0.0055 (8)	0.0041 (8)	0.0020 (8)
C16	0.0262 (10)	0.0267 (9)	0.0246 (9)	−0.0042 (8)	0.0082 (8)	−0.0023 (7)
C17	0.0308 (12)	0.0344 (11)	0.0489 (14)	0.0051 (9)	0.0108 (11)	0.0111 (10)

### Geometric parameters (Å, º)

**Table:**

I1—C4	2.106 (2)	C9—H9B	0.9800
S1—O3	1.4408 (14)	C9—H9C	0.9800
S1—O2	1.4410 (16)	C10—H10A	0.9800
S1—C1	1.738 (2)	C10—H10B	0.9800
S1—C11	1.7556 (19)	C10—H10C	0.9800
O1—C8	1.365 (2)	C11—C16	1.386 (3)
O1—C7	1.378 (2)	C11—C12	1.389 (3)
C1—C8	1.364 (3)	C12—C13	1.390 (3)
C1—C2	1.449 (3)	C12—H12	0.9500
C2—C7	1.387 (3)	C13—C14	1.385 (3)
C2—C3	1.395 (3)	C13—H13	0.9500
C3—C4	1.382 (3)	C14—C15	1.396 (3)
C3—H3	0.9500	C14—C17	1.504 (3)
C4—C5	1.394 (3)	C15—C16	1.387 (3)
C5—C6	1.391 (3)	C15—H15	0.9500
C5—H5	0.9500	C16—H16	0.9500
C6—C7	1.389 (3)	C17—H17A	0.9800
C6—C9	1.494 (3)	C17—H17B	0.9800
C8—C10	1.476 (3)	C17—H17C	0.9800
C9—H9A	0.9800		
			
O3—S1—O2	119.09 (9)	C6—C9—H9C	109.5
O3—S1—C1	106.23 (9)	H9A—C9—H9C	109.5
O2—S1—C1	108.99 (10)	H9B—C9—H9C	109.5
O3—S1—C11	108.50 (9)	C8—C10—H10A	109.5
O2—S1—C11	108.09 (9)	C8—C10—H10B	109.5
C1—S1—C11	105.09 (9)	H10A—C10—H10B	109.5
C8—O1—C7	106.93 (15)	C8—C10—H10C	109.5
C8—C1—C2	107.44 (17)	H10A—C10—H10C	109.5
C8—C1—S1	127.02 (16)	H10B—C10—H10C	109.5
C2—C1—S1	125.50 (15)	C16—C11—C12	121.08 (18)
C7—C2—C3	119.54 (18)	C16—C11—S1	120.01 (15)
C7—C2—C1	104.45 (17)	C12—C11—S1	118.86 (15)
C3—C2—C1	135.97 (18)	C11—C12—C13	118.69 (19)
C4—C3—C2	116.36 (18)	C11—C12—H12	120.7
C4—C3—H3	121.8	C13—C12—H12	120.7
C2—C3—H3	121.8	C14—C13—C12	121.37 (19)
C3—C4—C5	123.01 (19)	C14—C13—H13	119.3
C3—C4—I1	118.57 (15)	C12—C13—H13	119.3
C5—C4—I1	118.38 (15)	C13—C14—C15	118.84 (19)
C6—C5—C4	121.65 (19)	C13—C14—C17	120.4 (2)
C6—C5—H5	119.2	C15—C14—C17	120.8 (2)
C4—C5—H5	119.2	C16—C15—C14	120.7 (2)
C7—C6—C5	114.20 (19)	C16—C15—H15	119.7
C7—C6—C9	121.9 (2)	C14—C15—H15	119.7
C5—C6—C9	123.9 (2)	C11—C16—C15	119.33 (19)
O1—C7—C2	110.81 (17)	C11—C16—H16	120.3
O1—C7—C6	123.92 (18)	C15—C16—H16	120.3
C2—C7—C6	125.24 (19)	C14—C17—H17A	109.5
C1—C8—O1	110.37 (18)	C14—C17—H17B	109.5
C1—C8—C10	134.2 (2)	H17A—C17—H17B	109.5
O1—C8—C10	115.38 (18)	C14—C17—H17C	109.5
C6—C9—H9A	109.5	H17A—C17—H17C	109.5
C6—C9—H9B	109.5	H17B—C17—H17C	109.5
H9A—C9—H9B	109.5		
			
O3—S1—C1—C8	155.49 (18)	C9—C6—C7—O1	1.6 (3)
O2—S1—C1—C8	26.0 (2)	C5—C6—C7—C2	0.3 (3)
C11—S1—C1—C8	−89.6 (2)	C9—C6—C7—C2	179.4 (2)
O3—S1—C1—C2	−26.97 (19)	C2—C1—C8—O1	−0.2 (2)
O2—S1—C1—C2	−156.44 (16)	S1—C1—C8—O1	177.67 (14)
C11—S1—C1—C2	87.91 (18)	C2—C1—C8—C10	−179.7 (2)
C8—C1—C2—C7	−0.2 (2)	S1—C1—C8—C10	−1.8 (4)
S1—C1—C2—C7	−178.18 (15)	C7—O1—C8—C1	0.6 (2)
C8—C1—C2—C3	−177.5 (2)	C7—O1—C8—C10	−179.82 (19)
S1—C1—C2—C3	4.5 (3)	O3—S1—C11—C16	27.66 (18)
C7—C2—C3—C4	−1.0 (3)	O2—S1—C11—C16	158.10 (15)
C1—C2—C3—C4	176.0 (2)	C1—S1—C11—C16	−85.63 (17)
C2—C3—C4—C5	0.9 (3)	O3—S1—C11—C12	−154.88 (15)
C2—C3—C4—I1	−176.91 (14)	O2—S1—C11—C12	−24.44 (18)
C3—C4—C5—C6	−0.2 (3)	C1—S1—C11—C12	91.83 (16)
I1—C4—C5—C6	177.66 (16)	C16—C11—C12—C13	−1.2 (3)
C4—C5—C6—C7	−0.4 (3)	S1—C11—C12—C13	−178.63 (15)
C4—C5—C6—C9	−179.5 (2)	C11—C12—C13—C14	−0.3 (3)
C8—O1—C7—C2	−0.8 (2)	C12—C13—C14—C15	1.5 (3)
C8—O1—C7—C6	177.30 (19)	C12—C13—C14—C17	−178.7 (2)
C3—C2—C7—O1	178.48 (17)	C13—C14—C15—C16	−1.3 (3)
C1—C2—C7—O1	0.6 (2)	C17—C14—C15—C16	178.98 (19)
C3—C2—C7—C6	0.4 (3)	C12—C11—C16—C15	1.5 (3)
C1—C2—C7—C6	−177.42 (19)	S1—C11—C16—C15	178.85 (15)
C5—C6—C7—O1	−177.48 (18)	C14—C15—C16—C11	−0.2 (3)

### Hydrogen-bond geometry (Å, º)

**Table:**

*D*—H···*A*	*D*—H	H···*A*	*D*···*A*	*D*—H···*A*
C16—H16···O3^i^	0.95	2.58	3.246 (2)	127

Symmetry code: (i) −*x*+1, −*y*+1, −*z*+1.
